# The Synergism of the Small Molecule ENOblock and Fluconazole Against Fluconazole-Resistant *Candida albicans*

**DOI:** 10.3389/fmicb.2019.02071

**Published:** 2019-09-06

**Authors:** Liping Li, Teng Zhang, Jianrong Xu, Jing Wu, Yida Wang, Xiran Qiu, Yu Zhang, Weitong Hou, Lan Yan, Maomao An, Yuanying Jiang

**Affiliations:** ^1^Department of Pharmacology, Shanghai Tenth People’s Hospital, Tongji University School of Medicine, Shanghai, China; ^2^Department of Pharmacology and Chemical Biology, Institute of Medical Sciences, Shanghai Jiao Tong University School of Medicine, Shanghai, China; ^3^New Drug Research and Development Center, School of Pharmacy, Second Military Medical University, Shanghai, China; ^4^Department of Neurosurgery, Shanghai Tenth People’s Hospital, Tongji University School of Medicine, Shanghai, China

**Keywords:** *Candida albicans*, ENOblock, fluconazole, synergism, enolase1, transglutaminase activity

## Abstract

*Candida albicans* is the most common opportunistic fungal pathogen which can cause life-threatening bloodstream infections known as candidaemia. It is very important to discover new drugs and targets for the treatment of candidaemia. In this study, we first investigated the combination antifungal effects of the small molecule ENOblock and fluconazole (FLC) against FLC-resistant *C. albicans*. A checkerboard microdilution assay showed that ENOblock has a significant synergistic effect in combination with FLC against FLC-resistant *C. albicans*. The time-kill curve further confirmed the synergism of this compound with FLC against FLC-resistant *C. albicans*. Moreover, we demonstrated the significant inhibitory effects of ENOblock alone and in combination with FLC against *C. albicans* hypha and biofilm formation. Furthermore, the XTT assay showed that ENOblock has relatively low toxicity to human umbilical vein endothelial cells. The *in vivo* antifungal efficacy of ENOblock was further assessed in a murine model of systemic *C. albicans* infection. Although ENOblock alone was not sufficient to treat *C. albicans* infection, the combination of FLC and ENOblock showed significant *in vivo* activity against FLC-resistant *C. albicans*. Finally, using surface plasmon resonance analysis as well as an inhibition assay, we determined that ENOblock directly interacted with *Ca*Eno1 and significantly inhibited the transglutaminase activity of this enzyme, which is involved in the growth and morphogenesis of *C. albicans*. In summary, these results demonstrate the synergistic effects of FLC and ENOblock against FLC-resistant *C. albicans*, and indicate that inhibition of the transglutaminase activity of *Ca*Eno1 by ENOblock might confer an advantage for the synergism of FLC and ENOblock, suggesting the potential of ENOblock as a new antifungal candidate.

## Introduction

The treatment of invasive fungal infections with high mortality rates in immunocompromised hosts is notoriously difficult due to drug resistance and the limited number of antifungal agents available (Brown et al., 2012; Calderone et al., 2014; Ng et al., 2015). *Candida albicans*, one of the opportunistic fungal pathogens, which include non-*albicans Candida* species, *Cryptococcus neoformans* and *Aspergillus fumigatus*, is the most common cause of disseminated systemic candidiasis, especially in immunocompromised individuals, with mortality rates more than 40% (Pfaller and Diekema, 2007; Gow and Yadav, 2017; Robbins et al., 2017). Currently, the major classes of antifungals used in clinic are polyenes, azoles and echinocandins. However, the broad and irrational utilization of azoles, especially fluconazole (FLC), has led to the emergence azoles-resistant clinical isolates (White et al., 1998, 2002; Kohli et al., 2002). The treatment of *C. albicans* infection has been challenging due to the substantial host toxicities from current antifungal agents and the emergence of drug resistance to standard therapies (Perfect, 2016; Robbins et al., 2017). Moreover, *C. albicans* biofilms readily forming on medical devices show high levels of resistance to most of the antifungal agents and play a contributing role in the process of *C. albicans* infections (Ramage et al., 2009; Tobudic et al., 2010; Sarkar et al., 2014; Rajendran et al., 2015; Gow and Yadav, 2017). Antifungal resistance is a serious threat to human health, and there is an urgent need for the development of novel antifungals.

As a homolog of human enolase, enolase 1 (Eno1) encoded by the only one *ENO1* gene in *C. albicans* is a multifunctional protein that is essential for the growth and virulence of *C. albicans* (Yang et al., 2006; Ko et al., 2013). In particular, the heterozygosity or homozygosity of the *ENO1* gene may affect the growth of *C. albicans* under some conditions, decrease virulence in the host and increase the susceptibility to various drugs, such as FLC (Yang et al., 2006; Ko et al., 2013; Vyas et al., 2018).

*Ca*Eno1 is likely to be a good antifungal target. Transglutaminases (TGases) are widely distributed in plants, fungi and animals and are known to catalyze the formation of N-ε-(γ-glutamyl)-lysine amide bonds, resulting in covalent cross-links between proteins (Ruiz-Herrera et al., 1995). As previously reported in cell-free extracts of *Saccharomyces cerevisiae* and *C. albicans*, TGase activity is closely associated with cell wall assembly, and inhibition of the activity of this enzyme decreased the incorporation of proteins into the cell wall (Ruiz-Herrera et al., 1995; Iranzo et al., 2002). Recently, the multifunctional protein *Ca*Eno1 in the cell wall was identified as a TGase that is involved in cell growth, cell division, and morphogenesis (Reyna-Beltran et al., 2018). Moreover, as one virulence determinant of *C. albicans*, Hwp1 has previously been identified as a substrate for mammalian TGases (Staab et al., 1999), indicating the virtual interaction between Hwp1 and *Ca*Eno1. All of the above findings indicate that inhibition of multiple functions of *Ca*Eno1 may emerge as a promising strategy for enhancement of the efficacy of antifungals.

As the first reported enolase inhibitor, ENOblock is a non-substrate analog that directly binds human enolase to affect the non-glycolytic functions of this enzyme and has been revealed to possess some bioactivities, such as anti-tumor and anti-diabetic activities (Jung et al., 2013; Cho et al., 2017). However, the antimicrobial activity of ENOblock, such as antifungal activity, has not been reported to date. Thus, it would be interesting to speculate that ENOblock could exhibit significant activity against common human fungal pathogens such as *Candida* species and *C. neoformans*, and to further assess the interaction of ENOblock with potential fungal targets and the potential antifungal efficacy of ENOblock.

In this study, we first explored the *in vitro* and *in vivo* antifungal efficacy of small molecule ENOblock, and measured the cytotoxicity of this compound to evaluate its potential as an antifungal agent. We further assessed the interaction of ENOblock with *Ca*Eno1 and the ability to inhibit *Ca*Eno1 enzymatic activity, which might contribute to the synergism of FLC and ENOblock. To test the antifungal activity, we mainly investigated the synergistic effects of ENOblock and FLC against clinical *C. albicans* isolates, and used a murine model of systemic *C. albicans* infection to test the potential therapeutic efficacy of ENOblock in combination with FLC.

## Materials and Methods

### Strains and Agents

Thirty clinical isolates of FLC-resistant *C. albicans* including *C. albicans* strain 0304103, *C. albicans* reference strain SC5314, *C. neoformans* 56992, *C. tropicalis* ATCC20026, *C. parapsilosis* ATCC 22010, *C. krusei* ATCC2340, and *C. glabrata* ATCC1182, were used in this study. All of the clinical isolates including *C. albicans* strain 0304103 were provided by Changhai Hospital of Shanghai, the People’s Republic of China, and were widely used in our lab (Quan et al., 2006; Li et al., 2015a, b, 2017). *C. albicans* reference strain SC5314 can form normal biofilms and is susceptible to FLC (Ramage et al., 2001; Paulone et al., 2017). All of the various strains were grown in YPD (1% yeast extract, 2% peptone, and 2% dextrose) liquid medium overnight in a shaking incubator at 30°C. Human umbilical vein endothelial cells (HUVECs) were purchased from ATCC (Manassas, VA, United States) and incubated in Dulbecco modified Eagle medium (DMEM) containing 10% fetal bovine serum (FBS), and then were used to evaluate the toxicity of ENOblock (Beedie et al., 2016; Ma et al., 2016; Zhong et al., 2017).

Fluconazole (Sigma), and ENOblock hydrochloride (Selleck) were dissolved in dimethyl sulfoxide (DMSO), and the initial stock concentration was 6.4 mg/ml for susceptibility testing. For all experiments, DMSO comprised less than 1% of the total test volume to avoid affecting outcomes of experiments.

### Antifungal Susceptibility Testing

Antifungal susceptibility testing was performed according to the broth microdilution protocol of the Clinical and Laboratory Standards Institute (M27-A3), with a few modifications (Quan et al., 2006; Li et al., 2017; Zhong et al., 2017). Briefly, after 24 h or 72 h of incubation at 35°C, the growth inhibition was measured by the optical densities at 600 nm (OD_600_), from which the background optical densities were already subtracted. The final concentration of ENOblock and FLC ranged from 0.125 to 64 μg/ml, and then MICs were determined. Synergsim and antagonism were defined by FICIs of ≤ 0.5 and > 4, respectively. An FICI value of > 0.5 but ≤ 4 was considered indifferent (Odds, 2003).

### Time-Kill Test

*Candida albicans* 0304103 was grown in RPMI 1640 medium with a starting inoculum of 10^5^ CFU/ml. Different concentrations of ENOblock (8 μg/ml or 16 μg/ml) and FLC (8 μg/ml) were added. DMSO comprised < 1% of the total test volume. At predetermined time points (0, 4, 8, 12, 16, 24, 36, and 48 h) after incubation with agitation at 30°C, a 100-μl aliquot was removed from every solution and serially diluted 10-fold in PBS. A 100-μl aliquot from each dilution was spread on a SDA plate. Colony counts were determined after incubation at 30°C for 48 h. Synergism and antagonism were defined as described previously (Canton et al., 2005; Quan et al., 2006; Pankey et al., 2014). Briefly, Synergism was defined as a ≥ 2-log_10_ decrease in CFU per milliliter for the combination compared to that by the most active agent alone after 24 h, antagonism was defined as a ≥ 2-log_10_ increase in CFU per milliliter for the combination compared to that by the most active agent alone after 24 h, while a change of < 2 log_10_ CFU/ml was considered indifferent.

### Biofilm Formation and XTT Reduction Assay

The *in vitro* biofilm formation assay was carried out according to methods described previously (Ramage et al., 2002; Li et al., 2017) with some modifications. In brief, 100-μl aliquots of *C. albicans* 0304103 cells or *C. albicans* SC5314 (1.0 × 10^6^ CFU/ml) in RPMI 1640 medium were introduced into wells of 96-well tissue culture plates and incubated statically at 37°C. After 1 h of adhesion, the medium and non-adherent cells were removed, and the fresh RPMI 1640 medium was added. The plates were further incubated statically for 48 h at 37°C. Biofilm formation was semiquantitatively measured using a 2,3-bis(2-methoxy-4-nitro-5-sulfophenyl)-2H-terazolium-5-carboxanilide (XTT) reduction assay (Ramage et al., 2001). To investigate the effect of FLC in the absence or presence of ENOblock (2 to 16 μg/ml) on the formation of *C. albicans* biofilms, different concentrations of the agents were added to the fresh medium after 1 h of adhesion. Following incubation at 37°C for 48 h, biofilm cells were washed with PBS and incubated with 0.5 mg/ml XTT and 1 mM menadione in PBS at 37°C for 90 min. The optical density (OD) was measured at 490 nm using a microtiter plate reader. To detect the synergistic effect on mature biofilms, FLC and ENOblock (8 to 64 μg/ml) were added after 24 h of incubation with the mature biofilms at 37°C, and the plates were incubated at 37°C for an additional 24 h.

### Toxicity Evaluation of ENOblock

Human umbilical vein endothelial cells (HUVECs) were purchased from ATCC (Manassas, VA, United States) and incubated in DMEM containing 10% FBS (Beedie et al., 2016; Ma et al., 2016; Zhong et al., 2017). The cytotoxic effect of ENOblock on HUVECs viability was assessed by the XTT assay (Scudiero et al., 1988). HUVECs (5 × 10^3^ cells/well) were seeded in 96-well microtiter plates and cultured in DMEM medium (HyClone) supplemented with 10% FBS (HyClone) at 37°C for 24 h in the presence of 5% CO_2_. After incubation, the cell supernatant was removed and different concentrations of ENOblock (2 to 64 μg/ml) dissolved in fresh media without FBS were added. The plates were incubated for another 24 h. At the end of incubation, 50 μl of PMS-XTT solution (final concentration, 50 μg of XTT, and 0.38 μg of PMS per well) was added to each well and incubated at 37°C for 4 h. The absorbance at 450 nm was measured using an ELISA Plate Reader. Cells incubated in DMEM just with DMSO (<1% of the total test volume) were set as the standard for 100% viability.

The hemolytic activity of ENOblock was assessed using a previously reported method (Radwan et al., 2017; Yang et al., 2019) with some modifications. Briefly, 100 μl of a healthy rabbit red cell suspension in 96-well microtiter plates was mixed with 100 μl of a solution containing different concentrations of ENOblock, and final ENOblock concentrations of 2 to 64 μg/ml were applied. After 1 h of incubation at 37°C, the supernatants were collected, and the absorbance (Abs) at 405 nm was measured using a spectrophotometer. Additionally, red cells incubated with PBS containing DMSO (<1% of the total test volume) served as a negative-control group (to estimate natural hemolysis of PBS with DMSO), and those incubated with distilled water served as a positive control (serving as 100% hemolysis). The percentage of hemolysis induced by ENOblock was then calculated using the equation% hemolysis = (Abs_S_ – Abs_0_)/(Abs_100_ – Abs_0_) × 100%, where Abs_S_ is the average absorbance of the sample, Abs_0_ is the average absorbance of the negative control, and Abs_100_ is the average absorbance of the positive control. AMB and FLC were used as controls.

### Recombinant Protein Expression and Purification

The full-length *C. albicans* enolase 1 gene (*ENO1*, C1_08500C_A/orf19.395, *Candida* Genome Database) cloned into the pET-21a(+) plasmid with a C-terminal His_6_ tag was expressed in the *Escherichia coli* BL21(DE3)pLysS strain. For protein expression, cultures were grown in LB medium containing 0.1 mg/ml ampicillin in a rotary shaker at 37°C to an OD_600 nm_ of 0.6–0.8 before induced using 0.5 mM IPTG at 37°C for 5 h. After harvesting by centrifugation, cells were lysed by sonication in lysis buffer, and then centrifuged at more than 12,000 rpm for 20 min to collect the supernatant containing recombinant protein.

To obtain highly purified protein, the recombinant *C. albicans* enolase 1 (r*Ca*Eno1) protein was purified by Ni-chelate affinity chromatography (Qiagen), followed by size exclusion chromatography using a HiLoad 16/60 Superdex 200 prep grade column (GE Healthcare). Following purity determination by SDS-PAGE, aliquots (less than 100 μl) of purified recombinant proteins were flash-frozen with liquid nitrogen and stored at −80°C.

### Protein Activity Determination

The enolase activity of r*Ca*Eno1 was determined using the Enolase Activity Assay Kit (Sigma-Aldrich) according to the manufacturer’s instructions with slight modification. Briefly, the mixture with a final volume of 100 μl (50 μl of sample dilutions + 50 μl of the appropriate reaction mix) was mixed well by pipetting and incubated at 25°C. To test the inhibition of enolase activity by ENOblock, r*Ca*Eno1 was preincubated with ENOblock or sodium fluoride (NaF) in a total volume of 50 μl at room temperature, and then the experiment was continued as described above. After 30 min, the absorbance at 570 nm was measured as the OD_570 nm_ value. NaF used as a control is a well-known enolase inhibitor.

The transglutaminase activity of r*Ca*Eno1 was determined using Transglutaminase Assay Kit (Sigma) according to the manufacturer’s instructions. The kit can also be used to assay the inhibitory activity of ENOblock. Briefly, r*Ca*Eno1 and ENOblock were added into the appropriate wells, and the volume was adjusted to 50 μl with ultrapure water. r*Ca*Eno1 was preincubated with ENOblock or cystamine at room temperature, and then the experiment was continued according to the manufacturer’s instructions. The absorbance at 450 nm was measured as the OD_450 nm_ value. Cystamine was used as a control is a specific inhibitor of TGase.

### Surface Plasmon Resonance (SPR) Analysis

Surface plasmon resonance analysis was performed with a Biacore T200 instrument (GE Healthcare) with CM5 sensor chips (GE Healthcare). Activation, deactivation, preparation of the coupled flow cell and the ligand-binding assay were performed essentially as described previously (Song et al., 2016). Briefly, the r*Ca*Eno1 protein was immobilized in parallel-flow channels of on a BIAcore^TM^ CM5 sensor chip using an amine coupling kit (GE Healthcare). To test the binding of r*Ca*Eno1 protein by ENOblock, serial dilutions of ENOblock were injected into the flow system. Experiments were conducted with PBS as the running buffer, and the analyte was injected at a flow rate of 30 μl/min. The association time was 90 s and the dissociation time was 60 s. The affinity constants of binding were obtained using a 1:1 Langmuir binding model via BIAevaluation software. To avoid the non-specific protein binding, bovine serum albumin (BSA) was used as the negative control, and amphotericin B (AMB) which is big and greasy like ENOblock was used as the other control.

### Murine Model of Disseminated Candidiasis

After growth in YPD broth for 14 h at 30°C and 200 rpm, *C. albicans* 0304103 cells or *C. albicans* SC5314 cells were washed with PBS three times and diluted to the desired concentration (2.5 × 10^6^ cells/ml) determined by counting with a hemacytometer. Female C57BL/6 mice aged 8–10 weeks were infected via the tail vein with 200 μl of a 2.5 × 10^6^ CFU/ml PBS suspension, and then randomly placed into four groups (five mice per group): an untreated control group, FLC alone group, ENOblock alone group and FLC + ENOblock group. At 2 h after infection, 0.25 mg/kg FLC and 12 mg/kg ENOblock were administered intraperitoneally (i.p.). At 48 h following infection, the mice were euthanized and the kidneys were removed aseptically, placed in PBS, and then homogenized via bead beating. Serial dilutions of the homogenized kidneys were inoculated on YPD agar plates to measure the renal fungal burden. CFU values in kidneys were expressed as CFU/g of tissue, then transformed into log_10_ units and the differences between groups were analyzed by analysis of variance (ANOVA). For the survival assay (10 mice per group), FLC (0.25 mg/kg) and ENOblock (12 mg/kg) were administered i.p. at 2, 24, and 48 h post infection. Mice were given free access to standard laboratory diet and water, and checked daily until day 30. The survival curves were statistically analyzed by the Kaplan-Meier method (log-rank test, GraphPad Prism).

### Statistical Criteria

Statistics were calculated in GraphPad Prism 6.0 (GraphPad Software, San Diego, CA, United States), in which *P*-value of < 0.05 (^∗^), < 0.01 (^∗∗^), or < 0.001 (^∗∗∗^) was considered statistically significant.

## Results

### *In vitro* Synergistic Activity of FLC and ENOblock Against FLC-Resistant *C. albican*s

ENOblock is the first reported non-substrate inhibitor of enolase. Here, we first evaluated the antifungal activity of ENOblock alone or in combination with FLC against two *C. albicans* isolates (FLC-sensitive *C. albicans* SC5314 and FLC-resistant *C. albicans* 0304103) and various other yeast strains, including *C. neoformans, Candida krusei*, *Candida tropicalis*, *Candida glabratas*, and *Candida parapsilosis* (Table 1). The MICs of ENOblock alone against these tested strains ranged from 8 to 64 μg/ml. When ENOblock was used in combination with FLC, the MICs ranged from 1 to 16 μg/ml. In terms of MICs, the synergistic activity was observed in FLC-resistant *C. albicans* 0304103, *C. krusei*, *C. glabratas*, and *C. parapsilosis*. However, the combination of FLC and ENOblock did not show synergism against the FLC-susceptible *C. albicans* SC5314, exhibiting a fractional inhibitory concentration index (FICI) of 1.03 (Table 1).

**TABLE 1:** Interaction of fluconazole (FLC) and ENOblock against various yeast species.

**Yeast strains**	**MICs (μg/ml) alone**		**MICs (μg/ml) in combination**		**FICI**	**Mode of interaction**
	**FLC**	**ENOblock**	**FLC**	**ENOblock**		
*C. albicans* SC5314	1	32	1	1	1.03	Indiff
*C. albicans* 0304103	>64	32	2	8	0.27	Syn
*C. neoformans* 56992	4	32	0.125	16	0.52	Indiff
*C. krusei* ATCC2340	64	64	16	8	0.38	Syn
*C. tropicalis* ATCC20026	2	8	0.5	2	0.50	Indiff
*C. glabrata* ATCC1182	8	16	0.5	1	0.13	Syn
*C. parapsilosis* ATCC22019	2	64	0.125	16	0.31	Syn

*FLC, fluconazole; Syn, synergism; Indiff, indifference; FICI < 0.5, Syn; 0.5 < FICI < 4, Indiff.*

To further assess the antifungal activity of ENOblock against *C. albicans*, we tested the effect of ENOblock in combination with FLC against 30 clinical FLC-resistant and 10 FLC-sensitive *C. albicans* isolates by a checkerboard microdilution assay. The results are summarized in Table 2. The range of MICs of FLC alone against all the FLC-resistant strains was > 64 μg/ml, and the MICs of ENOblock alone ranged from 16 to 32 μg/ml. Compared to FLC alone, the combination of FLC and ENOblock markedly reduced the MICs and showed a significant synergistic effect against all the FLC-resistant strains tested, as the FICI ranged from 0.13 to 0.38 (Table 2). Unlike the results of FLC-resistant isolates, the combination of FLC and ENOblock did not exhibit synergism on FLC-susceptible strains tested, since the FICI was 0.56 to 1.06 (Table 2).

**TABLE 2:** Interaction of fluconazole (FLC) and ENOblock against *C. albicans* as determined by microdilution assay^a^.

**Parameter**	**MICs (μg/ml)**					
	**FLC-resistant *C. albicans* (*n* = 30)**		**FLC-sensitive *C. albicans* (*n* = 10)**	
	**FLC**	**ENOblock**	**FLC**	**ENOblock**
Agent alone	>64	16–32	0.5–1	16–32
Agent combination (FLC/ENOblock)	0.5–32/2–8		0.25–1/1	
FICI	0.13–0.38		0.56–1.06	

*^*a*^Detailed MICs data are presented in Supplementary Tables S1, S2 in the Supplementary Material.*

**FIGURE 1:**
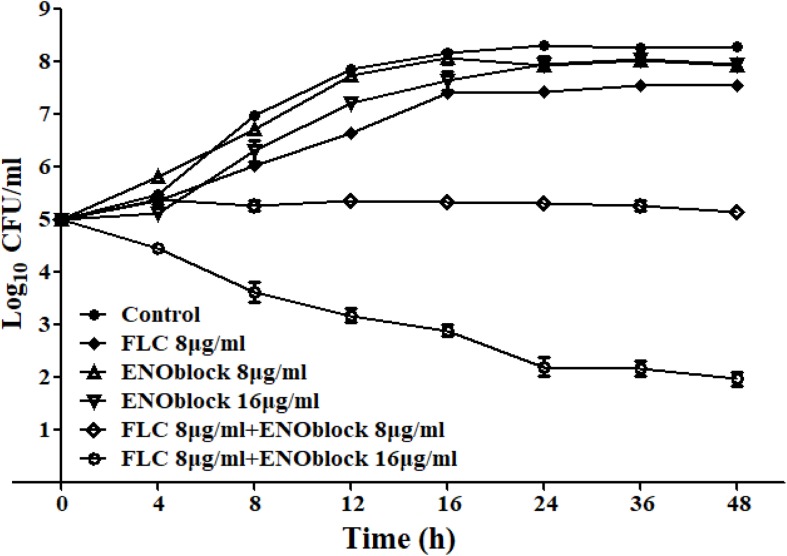
Time-kill curves of *Candida albicans* 0304103 treated with different concentrations of ENOblock/FLC using an initial inoculum of 10^5^ CFU/ml. Aliquots were obtained at the indicated time points and serially dilutions were spread on agar plates. Colony counts were determined after 48 h of incubation. The data represent the means ± standard deviations for three independent experiments.

In addition, the time-kill assay also confirmed the synergism of ENOblock and FLC against FLC-resistant *C. albicans* 0304103 (Figure 1). As shown in Figure 1, compared with the control with a starting inoculum of 10^5^ CFU/ml, ENOblock alone, even at 16 μg/ml, had no impact on the viability of *C. albicans* 0304103 after 24 h, while FLC (8 μg/ml) alone had a weak effect on its growth. However, the combination of ENOblock (8 μg/ml) and FLC (8 μg/ml) produced a 2.1-log10-CFU/ml decrease compared with the number of CFU produced by FLC (8 μg/ml) alone at 24 h, suggesting the combination of ENOblock and FLC significantly enhanced the antifungal effect compared with FLC (8 μg/ml) alone. Meanwhile, the antifungal effect was further improved as the dose (16 μg/ml) of ENOblock increased when the dose (8 μg/ml) of FLC remained unchanged (Figure 1), indicating that the synergistic activity of ENOblock and FLC was dependent on the dose of ENOblock.

### Effects of ENOblock Against *C. albicans* Hypha and Biofilm Formation

**FIGURE 2:**
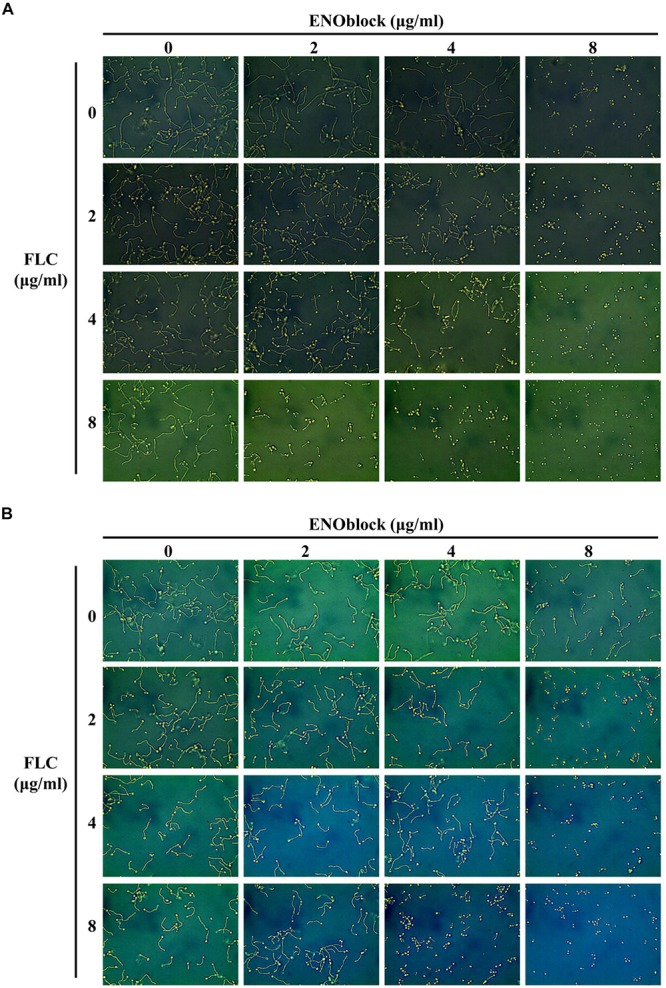
Effects of different concentrations of ENOblock in combination with FLC on hypha formation. Representative photomicrographs of indicated *C. albicans* 0304103 growing in different media, including liquid Lee medium **(A)** and liquid Spider medium **(B)**, for 3.5 h at 37°C, as observed with an inverted phase contrast microscope with a 40 × objective.

To determine the effect of ENOblock alone or in combination with FLC on the yeast-to-hypha morphological transition of *C. albicans*, *C. albicans* 0304103 cells were grown in several different media that are known to induce morphological transition, including liquid Lee medium, liquid Spider medium and liquid RPMI 1640 medium. In all the ENOblock-free media with or without FLC (2 to 8 μg/ml), *C. albicans* 0304103 cells formed true hyphae (Figure 2 and Supplementary Figure S1). Interestingly, 4 μg/ml ENOblock in combination with FLC (2 to 8 μg/ml) inhibited the yeast-to-hypha morphological transition to some extent in liquid Lee or Spider medium, and the inhibitory effect was dependent on the dose of ENOblock. The addition of 8 μg/ml ENOblock obviously disrupted the formation of true hyphae: pseudohyphae and yeast-form cells were observed in liquid Lee or Spider medium without FLC, and hypha formation was completely inhibited in liquid Lee and Spider media by 8 μg/ml ENOblock in combination with FLC (2 to 8 μg/ml) (Figure 2 and Supplementary Figure S1). However, in liquid RPMI 1640 medium with ENOblock (8 μg/ml) plus FLC (2 to 8 μg/ml), the morphological transition began to be inhibited (Supplementary Figure S2). When in combination with FLC (2 to 8 μg/ml), ENOblock at 16 μg/ml substantially damaged the formation of true hyphae, and only yeast-form cells were observed in liquid RPMI 1640 medium when the ENOblock concentration approached 32 μg/ml (Supplementary Figure S2).

**FIGURE 3:**
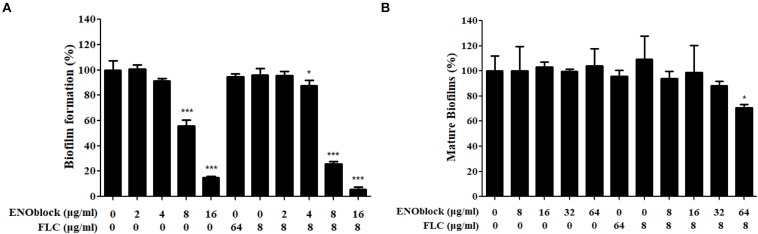
Effects of the combination of ENOblock and FLC on biofilm formation of *C. albicans* 0304103 *in vitro*. **(A)** Effects of different concentrations of ENOblock alone and in combination with 8 μg/ml FLC on *C. albicans* biofilm formation. **(B)** Effects of different concentrations of ENOblock alone and in combination with 8 μg/ml FLC on the maintenance of mature biofilms. Biofilm formation was evaluated by the XTT reduction assay by calculating the percentage of viable *C. albicans* cells relative to the control cells without drug treatment. Data are shown as the means ± standard deviations for three independent experiments. ^∗∗^*P* < 0.01; ^∗∗∗^*P* < 0.001 compared with the value of the control biofilms.

Furthermore, the *in vitro* inhibitory activity of ENOblock alone or in combination with FLC against *C. albicans* biofilm formation was assessed by the XTT reduction assay. As shown in Figure 3A, 8 μg/ml and 16 μg/ml ENOblock inhibited biofilm formation by approximately 45 and 80%, respectively (*P* < 0.001), while FLC used alone at both 8 and 64 μg/ml did not exhibit antibiofilm activity. Notably, at 4 μg/ml, ENOblock alone had no effect on biofilm formation, but in combination with FLC (8 μg/ml), ENOblock exhibited a significant inhibitory effect with an of approximately 12.5% reduction of biofilm formation (*P* < 0.05). The inhibitory activity increased when the ENOblock concentration increased. The combination of ENOblock (8 μg/ml or 16 μg/ml) and FLC (8 μg/ml) substantially reduced biofilm formation by approximately 75% or 95%, respectively (*P* < 0.001). Specifically, the combination of ENOblock and FLC exhibited inhibitory activity against mature biofilms. Neither ENOblock nor FLC alone had a significant inhibitory effect on mature biofilms (Figure 3B), but ENOblock (64 μg/ml) in combination with FLC (8 μg/ml) destroyed mature biofilms by 29% (*P* < 0.05). Consistently, as shown in Supplementary Figure S3, the significant inhibitory activity of ENOblock against biofilm formation of *C. albicans* SC5314 was also observed and even more obvious.

### The *in vivo* Activity of ENOblock-FLC Combination in a Murine Model of Systemic Candidiasis

**FIGURE 4:**
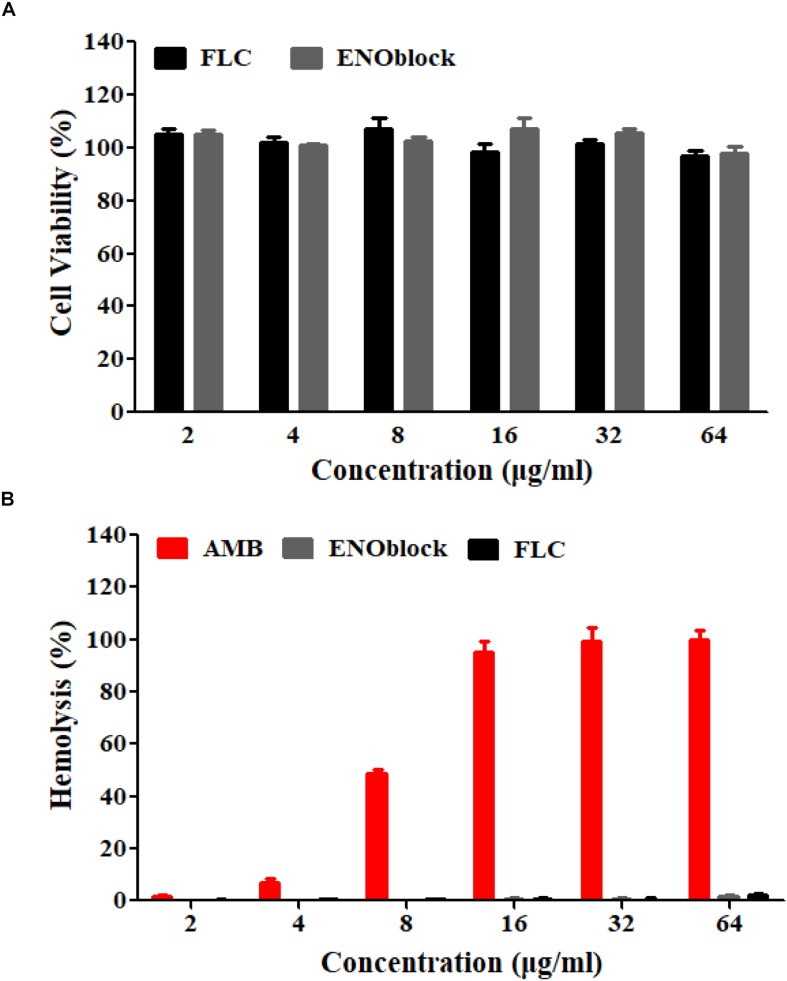
The *in vitro* toxicity evaluation of ENOblock. **(A)** The cytotoxic effect of ENOblock, compared to that of FLC, on HUVEC viability was assessed by the XTT test following a 4-h treatment. **(B)** Hemolysis of rabbit red blood cells following incubation with ENOblock. AMB and FLC were used as controls. The results represent the means ± standard deviations from three independent experiments.

**FIGURE 5:**
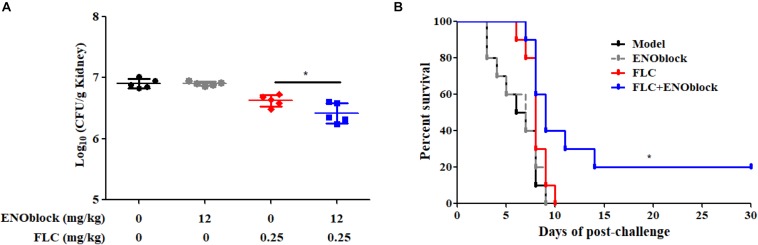
ENOblock enhances the activity of FLC in a murine model of systemic candidiasis. **(A)** Kidney CFU assay in mice with systemic candidiasis after 2 days. Female C57BL/6 mice were infected with 5 × 105 CFU of *C. albicans* 0304103. ENOblock and FLC were administered 2 h post infection. ^∗^*P* < 0.05 (*P*-values are from ANOVA). **(B)** Survival curves of C57BL/6 mice infected with 5 × 105 CFU of *C. albicans* 0304103. ENOblock and FLC were administered at 2, 24, and 48 h post infection. The log-rank test was used for statistical analysis.

To evaluate the efficacy of combination therapy with FLC and ENOblock, we compared the renal fungal burden and survival rate of *C. albicans*-infected mice using the well-established murine model of systemic candidiasis. Firstly, we checked the *in vitro* toxicity of ENOblock including cell viability and hemolysis, and found that ENOblock had neither significant toxic effect on HUVECs nor *in vitro* hemolytic activity at concentrations that show significant antifungal activity (Figure 4). In a murine model of systemic candidiasis with *C. albicans* 0304103, treatment of mice with ENOblock (12 mg/kg) alone did not show significant reduction in fungal burden when compared to the untreated counterparts. However, the combination of ENOblock and FLC significantly reduced the renal fungal burden and improved the survival relative to FLC alone (^∗^*P* < 0.05) (Figure 5). Consistent with this finding, the combination treatment also significantly reduced the renal fungal burden and extended the survival of *C. albicans* SC5314-infected mice, while neither FLC nor ENOblock was sufficient to treat the *C. albicans* infection (Supplementary Figure S4), confirming the synergistic *in vivo* antifungal efficacy of these two agents.

### Effects of ENOblock on the Transglutaminase Activity of *C. albicans* Enolase1

It has been reported that ENOblock binds to purified human enolase and inhibits the activity of the enzyme (Jung et al., 2013). In this study, we cloned the full-length *C. albicans* enolase 1 gene (*ENO1*, C1_08500C_A/orf19.395, *Candida* Genome Database) into the pET-21a(+) vector with a C-terminal His_6_ tag and purified recombinant *C. albicans* enolase 1 (r*Ca*Eno1) produced in *E. coli* BL21(DE3)pLysS using a Ni^2+^-NTA-agarose column. The r*Ca*Eno1 protein was identified by Western blotting with anti-His-tag antibodies and mouse anti-r*Ca*Eno1 antibodies produced with purified r*Ca*Eno1 (Supplementary Figure S5).

**FIGURE 6:**
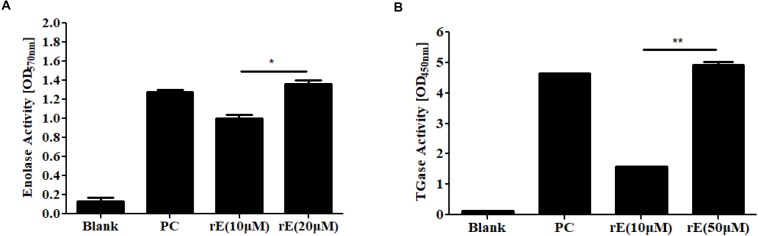
Enzyme activity of recombinant *C. albicans* enolase 1 (r*Ca*Eno1). **(A)** Enolase activity was determined with purified rCaEno1 protein. rE, rCaEno1; PC, enolase-positive control (catalog number MAK178F). **(B)** TGase activity was determined with purified rCaEno1 protein. rE, rCaEno1; PC, positive control (transglutaminase from guinea pig liver, catalog number T5398). ^∗^*P* < 0.05; ^∗∗^*P* < 0.01 (statistical two-tailed unpaired *t*-test). Data are shown as the means ± standard deviations for three independent experiments.

**FIGURE 7:**
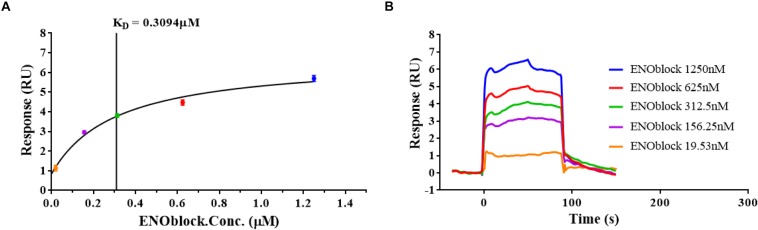
ENOblock binds recombinant *C. albicans* enolase 1 (r*Ca*Eno1). Interactions of r*Ca*Eno1 with ENOblock measured by surface plasmon resonance. The r*Ca*Eno1 protein was coated on the CM5 sensor chip and serial dilutions of ENOblock (1250, 625, 312.5, 156.25, and 19.53 nM) were used as analytes. Changes in plasmon resonance are shown as response units. **(A)** The results of the steady-state affinity analysis (K_*D*_, equilibrium). Plotted in the left panel (colored circles) is the binding response (RU) versus concentration of ENOblock offered, and the vertical line indicates the binding affinity. **(B)** Binding curves (colored lines) obtained by passing different concentrations of ENOblock (1250, 625, 312.5, 156.25, and 19.53 nM) over r*Ca*Eno1 immobilized on a biosensor surface. The data are representative of two independent experiments.

Furthermore, we demonstrated the enolase activity and transglutaminase (TGase) activity of r*Ca*Eno1 using enzyme assay kits (Figures 6A,B), and investigated the binding of ENOblock to r*Ca*Eno1 using surface plasmon resonance (SPR), showing the potent interaction of them with a K_*D*_ of approximately 0.3 μM (Figure 7). To avoid the problem that this is just not non-specific protein binding, we also performed the SPR analysis using BSA and AMB as controls. The results indicated that while ENOblock showed strong interaction with rCaEno1p, it had no obvious binding with BSA. However, AMB showed weak binding with both r*Ca*Eno1p and BSA, suggesting the non-specific protein binding between AMB and r*Ca*Eno1p (Supplementary Figure S6).

**FIGURE 8:**
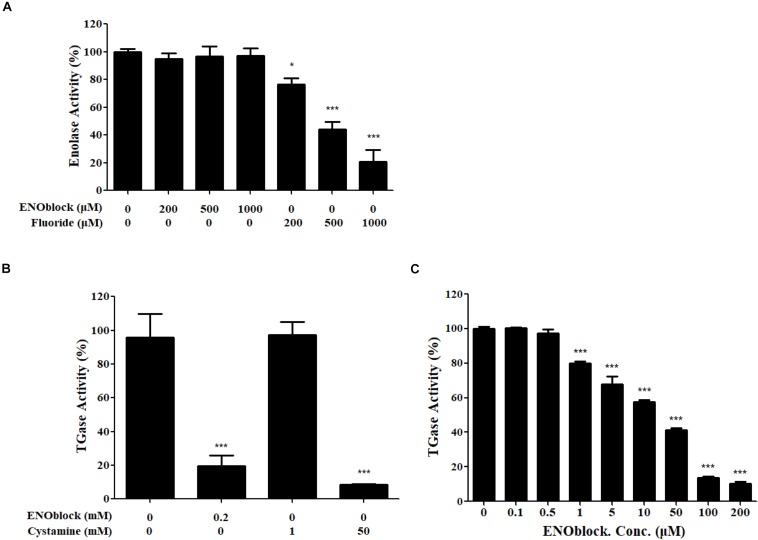
ENOblock inhibits the TGase activity of recombinant *C. albicans* enolase 1 (r*Ca*Eno1). **(A)** Inhibition of enolase activity was determined with purified rCaEno1 protein with or without different concentrations of ENOblock or sodium fluoride (NaF). The well-known enolase inhibitor NaF was used as a control. **(B)** Inhibition of TGase activity was determined with purified rCaEno1 protein with ENOblock or the specific inhibitor cystamine used as a control. **(C)** Inhibition of TGase activity was determined with purified rCaEno1 protein with or without different concentrations of ENOblock. ^∗^*P* < 0.05; ^∗∗∗^*P* < 0.001 compared to the untreated group (statistical two-tailed unpaired *t*-test). Data are shown as the means ± standard deviations for three independent experiments.

We then examined the inhibitory effect of ENOblock on the activities of r*Ca*Eno1 using the kits. As shown in Figure 8A, the well-known enolase inhibitor sodium fluoride (NaF) dose-dependently inhibited the enolase activity of r*Ca*Eno1, while ENOblock did not have a significant effect on it. Intriguingly, the TGase activity of r*Ca*Eno1 was significantly inhibited by ENOblock (Figure 8B), and this inhibition was dose dependent, as reflected by a progressive reduction in TGase activity with increasing concentrations of ENOblock (Figure 8C). The half maximal inhibitory concentration (IC_50_) of TGase inhibition by ENOblock is approximately 12.6 μM (7.95 μg/ml). Therefore, it indicated that the inhibition of TGase activity of *Ca*Eno1 by ENOblock resulted in the disruption of hypha and biofilm formation, and affected the cell wall construction of *C. albicans*, increasing the susceptibility of *C. albicans* to FLC and contributing to the synergism of FLC and ENOblock.

## Discussion

In this study, we focused on evaluating the potential of ENOblock as an antifungal drug by examining its antifungal activities against *C. albicans*, *C. neoformans*, and non-*albicans Candida*species, including *C. krusei*, *C. tropicalis*, *C. glabratas* and *C. parapsilosis* (Tables 1, 2). ENOblock in combination with FLC showed significant synergistic efficacy against FLC-resistant *C. albicans* after screening all 30 selected clinical FLC-resistant *C. albicans* strains. Whereas, FLC and ENOblock did not exhibit synergistic activity against ten FLC-sensitive *C. albicans* strains tested, clinical *C. neoformans* 56992, and *C. tropicalis* ATCC20026 (Tables 1, 2). This finding suggests that their synergistic antifungal activity might be relevant to FLC resistance, or that FLC alone was sufficiently effective against drug-sensitive strains at low concentrations, so the synergism could not be observed. In contrast, FLC alone was not enough for drug-resistant strains, so the synergism was discovered, which is consistent with the hypothesis proposed by Li et al. (2017).

Although, we focused on the antifungal activity of ENOblock alone or in combination with FLC against FLC-resistant *C. albicans* in this study, biofilm formation assay and antifungal therapy in the murine model were also examined on FLC-sensitive *C. albicans* SC5314 that is a reference strain which can form normal biofilms. ENOblock alone or in combination with FLC showed significant inhibitory activity against biofilm formation of *C. albicans* SC5314 (Supplementary Figure S3), and the combination of ENOblock and FLC also showed *in vivo* activity against *C. albicans* SC5314 although *in vitro* synergy measured by antifungal susceptibility test was not observed (Supplementary Figure S4). According to previous reports (Minn et al., 1997; Zhanel et al., 2001), it is known that *in vitro* testing of some compounds can be used to predict *in vivo* efficacy, but also may not correlate with *in vivo* efficacy, due to the influence of serum, formulation of compounds, and some other factors. It is possible to observe the *in vivo* synergism against *C. albicans* SC5314 in the animal study.

Enolase 1 was identified as a multifunctional protein showing non-glycolytic functions and is highly conserved across species, including plants, bacteria, and mammals (such as humans). *Ca*Eno1 interacts with some proteins of the glycolytic pathway (STRING database) and is also reported to be genetically associated with *C. albicans* Cbk1 (cell wall biosynthesis kinase), which is involved in regulating cell wall biosynthesis, hyphal morphogenesis and biofilm formation (McNemar and Fonzi, 2002; Song et al., 2008; Gutierrez-Escribano et al., 2012). Some virulence determinants such as Hwp1 in *C. albicans* cell wall have been shown to serve as acceptors of TGases (Sundstrom, 1999), suggesting that further exploration for targets of *Ca*Eno1 as a TGase should be an interesting area.

In addition, *Ca*Eno1 is described as an immunodominant antigen that is abundant predominantly in patients with invasive candidiasis and may be used as a biomarker or to produce candidate vaccines for the diagnosis and treatment of invasive candidiasis (Pitarch et al., 2014; Marin et al., 2015). As an integral protein of the cell wall, *Ca*Eno1 is responsible for TGase activity, but was also found in the secretome and extracellular vesicles (EVs) of *C. albicans* via different proteomic approaches, while its function in the secretome and EVs is still unclear (Gil-Bona et al., 2017).

As the first reported non-substrate small-molecule inhibitor of enolase, ENOblock is well tolerated in mice and has been revealed to inhibit cancer cell metastasis *in vivo*, and have potential anti-diabetic effects in wild-type zebrafish larvae and mammals via the inhibition of non-glycolytic functions of enolase, suggesting ENOblock is a suitable drug candidate for cancer and diabetes (Jung et al., 2013; Cho et al., 2017). In this study, our data confirmed that ENOblock in combination with FLC changed the fungistatic azoles into fungicidal (Figure 1), reversing antifungal drug resistance and broadening the therapeutic implications of combination therapy with azoles. In addition, ENOblock binds r*Ca*Eno1 with strong enzymatic activities as described above, and significantly inhibits its TGase activity, possibly contributing to the synergism of ENOblock and FLC. Furthermore, the XTT reduction assay showed the inhibitory effect of ENOblock alone or in combination with FLC against *C. albicans* biofilm formation, indicating that the binding of ENOblock to *Ca*Eno1 might impact interactions between *Ca*Eno1 and several important proteins associated with biofilm formation, such as Cbk1 and Hwp1. Although ENOblock has previously been shown to bind human enolase and inhibit its enolase activity, we did not observe any obvious difference in the enolase activity of r*Ca*Eno1 in the presence or absence of ENOblock at different concentrations, possibly because ENOblock acts via a mechanism other than direct inhibition of the enolase activity in different organisms (Satani et al., 2016). This finding could also suggest that the conformational difference between r*Ca*Eno1 and human enolase may result in no-detectable inhibitory effect of ENOblock on the enolase activity of r*Ca*Eno1. The further underlying mechanism for the antifungal effect of ENOblock binding *Ca*Eno1, using techniques such as fluorescent probe and the X-ray crystallography, should be worth carrying out.

## Conclusion

In conclusion, our present results demonstrate for the first time that ENOblock could enhance the antifungal activity of FLC against FLC-resistant *C. albicans in vitro*, and significantly improve the activity of FLC in a murine model of systemic candidiasis, while it alone has no therapeutic activity. Furthermore, our data suggest that the synergism of FLC and ENOblock was associated with the disruption of hypha and biofilm formation due to the inhibition of TGase activity of *Ca*Eno1 bound by ENOblock, and the potential of ENOblock as a new antifungal candidate. Next, we will carry out the modification and reconstruction of ENOblock as a pro-compound to produce several promising synergists in combination with FLC.

## Data Availability

The raw data supporting the conclusions of this manuscript will be made available by the authors, without undue reservation, to any qualified researcher.

## Ethics Statement

All animal experiments were performed under the standardized procedures of the “Regulations on the Administration of Laboratory Animals” approved by the State Council of the People’s Republic of China. The animal experimental protocol has been verified and approved by the Animal Care and Use Committee of Tongji University. All of the clinical isolates were provided by the Changhai Hospital of Shanghai, China.

## Author Contributions

MA and YJ conceived and designed the experiments. LL, TZ, JX, JW, YW, XQ, YZ, WH, and LY performed the experiments. LL and MA analyzed the data. LL wrote the manuscript.

## Conflict of Interest Statement

The authors declare that the research was conducted in the absence of any commercial or financial relationships that could be construed as a potential conflict of interest.

## Supplementary Material

FIGURE S1 Effects of different concentrations of ENOblock in combination with FLC on hypha formation in liquid Lee medium and liquid Spider medium. Representative photomicrographs of indicated *C. albicans* 0304103 growing in different media including liquid Lee medium **(A)** and liquid Spider medium **(B)** with different concentrations of ENOblock (0, 8, 16, and 32 μg/ml) and FLC (0, 2, 4, and 8 μg/ml), for 3.5 h at 37°C, as observed with an inverted phase contrast microscope with a 40 × objective.

FIGURE S2 Effects of different concentrations of ENOblock in combination with FLC on hypha formation in liquid RPMI 1640 medium. Representative photomicrographs of indicated *C. albicans* 0304103 grown in liquid RPMI 1640 medium for 3.5 h at 37°C, as observed with an inverted phase contrast microscope with a 40 × objective.

FIGURE S3 Effects of the combination of ENOblock and FLC on biofilm formation of *C. albicans* SC5314 *in vitro*. **(A)** Effects of different concentrations of ENOblock alone and in combination with 8 μg/ml FLC on *C. albicans* biofilm formation. **(B)** Effects of different concentrations of ENOblock alone and in combination with 8 μg/ml FLC on the maintenance of mature biofilms. Biofilm formation was evaluated by the XTT reduction assay by calculating the percentage of viable *C. albicans* cells relative to the control cells without drug treatment. Data are shown as the means ± standard deviations for three independent experiments. ^∗∗^*P* < 0.01; ^∗∗∗^*P* < 0.001 compared with the value of the control biofilms.

FIGURE S4 ENOblock enhances the activity of FLC in a murine model of systemic candidiasis. **(A)** Kidney CFU assay in mice with systemic candidiasis after 2 days. Female C57BL/6 mice were infected with 5 × 10^5^ CFU of *C. albicans* SC5314. ENOblock and FLC were administered 2 h post infection. ^∗^*P* < 0.05 (*P*-values are from ANOVA). **(B)** Survival curves of C57BL/6 mice infected with 5 × 10^5^ CFU of *C. albicans* SC5314. ENOblock and FLC were administered at 2, 24, and 48 h post infection. The log-rank test was used for statistical analysis.

FIGURE S5 Expression and purification of recombinant *C. albicans* enolase 1 (r*Ca*Eno1). **(A)** The r*Ca*Eno1 protein was purified by Ni^2+^-NTA-agarose column chromatography as described, and elution fractions were evaluated by 10% SDS-PAGE. Lane 1, protein marker; lane 2, negative control (empty vector); lane 3, whole-cell protein; lane 4, supernatant fractions; lane 5, precipitation fractions; lanes 6–8, washed fractions; lane 9, purified r*Ca*Eno1 fusion protein. **(B)** Western blot of total proteins from *C. albicans* SC5314 (lane 2) and purified r*Ca*Eno1 protein (lane 3) using mouse anti-r*Ca*Eno1 protein antibodies and anti-His-tag polyclonal antibodies (lane 4).

FIGURE S6 The interactions of r*Ca*Eno1 with ENOblock and amphotericin B measured by surface plasmon resonance. The r*Ca*Eno1 protein (BSA as the negative control) was coated on the CM5 sensor chip and serial dilutions (1562.5, 781.25, 390.6, 195.3, and 97.6 nM) of ENOblock (AMB as the control) were used as analytes. Changes in plasmon resonance are shown as response units. The panels **(A,B)** show binding curves (colored lines) obtained by passing different concentrations of ENOblock (1562.5, 781.25, 390.6, 195.3, and 97.6 nM) over r*Ca*Eno1 or BSA immobilized on a biosensor surface. The panels **(C,D)** show binding curves (colored lines) obtained by passing different concentrations of AMB (1562.5, 781.25, 390.6, 195.3, and 97.6 nM) over r*Ca*Eno1 or BSA immobilized on a biosensor surface. The data are representative of two independent experiments. AMB, amphotericin B.

TABLE S1 Interaction of fluconazole (FLC) and ENOblock against 30 FLC-resistant *C. albicans.*

TABLE S2 Interaction of fluconazole (FLC) and ENOblock against 10 FLC-sensitive *C. albicans*.
