# Supplementary figures and images for: The Synergism of the Small Molecule ENOblock and Fluconazole Against Fluconazole-Resistant Candida albicans
Source: Front Microbiol. 2019 Sep 6;10:2071. doi: 10.3389/fmicb.2019.02071 (PMC6742966; doi:10.3389/fmicb.2019.02071)

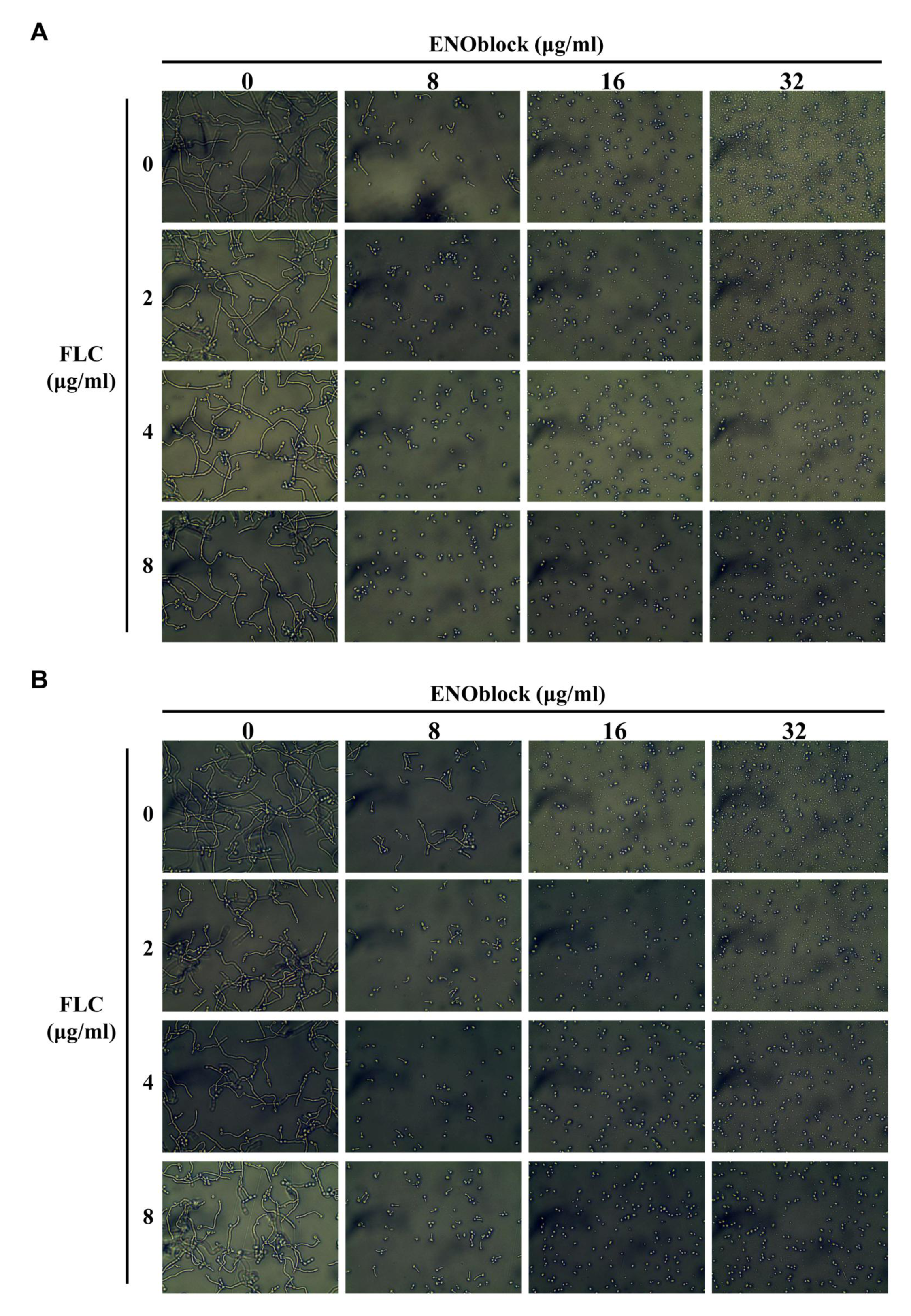

Supplement: FIGURE S1 — Effects of different concentrations of ENOblock in combination with FLC on hypha formation in liquid Lee medium and liquid Spider medium. Representative photomicrographs of indicated C. albicans 0304103 growing in different media including liquid Lee medium (A) and liquid Spider medium (B) with different concentrations of ENOblock (0, 8, 16, and 32 μg/ml) and FLC (0, 2, 4, and 8 μg/ml), for 3.5 h at 37°C, as observed with an inverted phase contrast microscope with a 40 × objective. [file Image_1.TIF]

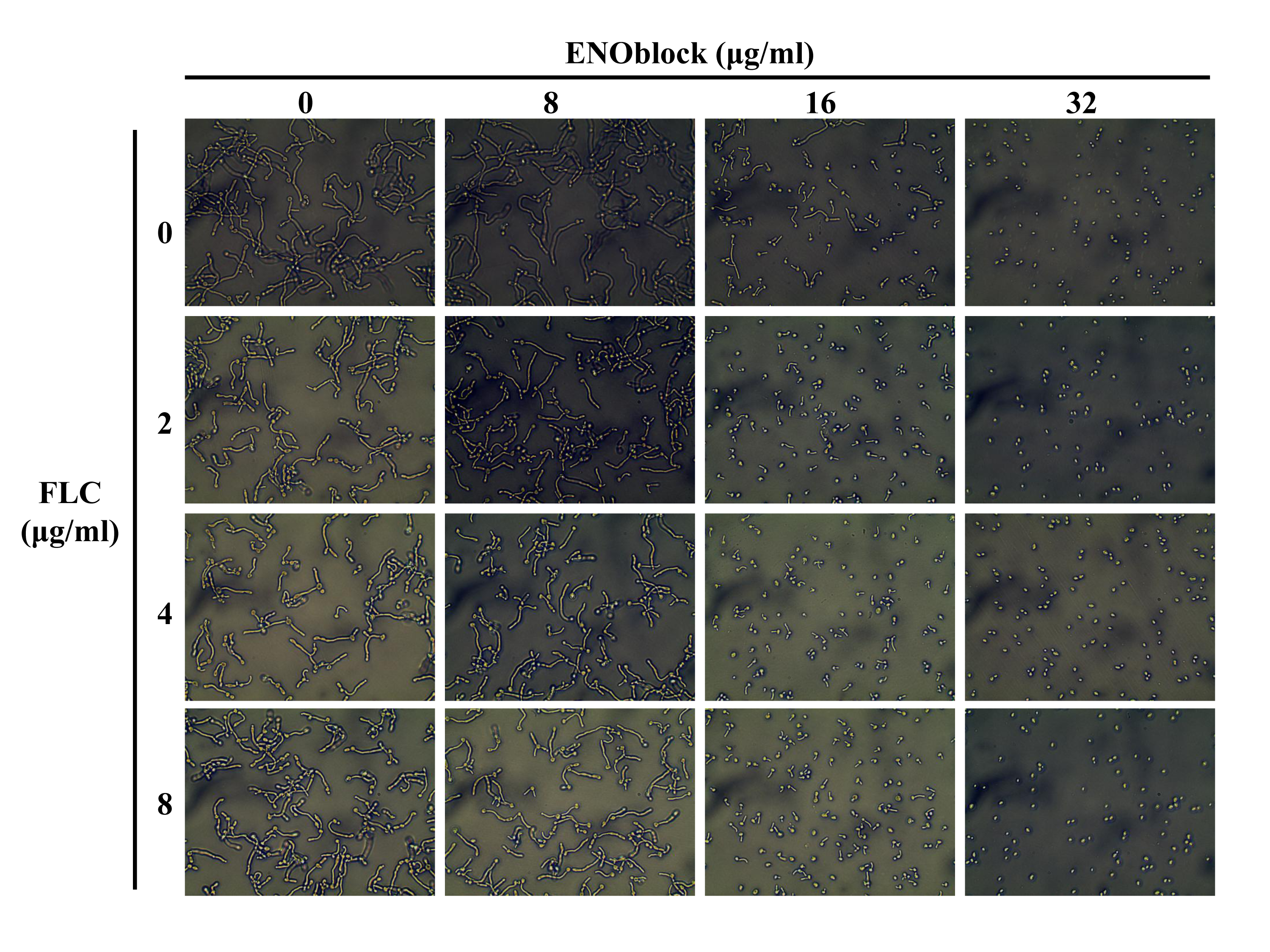

Supplement: FIGURE S2 — Effects of different concentrations of ENOblock in combination with FLC on hypha formation in liquid RPMI 1640 medium. Representative photomicrographs of indicated C. albicans 0304103 grown in liquid RPMI 1640 medium for 3.5 h at 37°C, as observed with an inverted phase contrast microscope with a 40 × objective. [file Image_2.TIF]

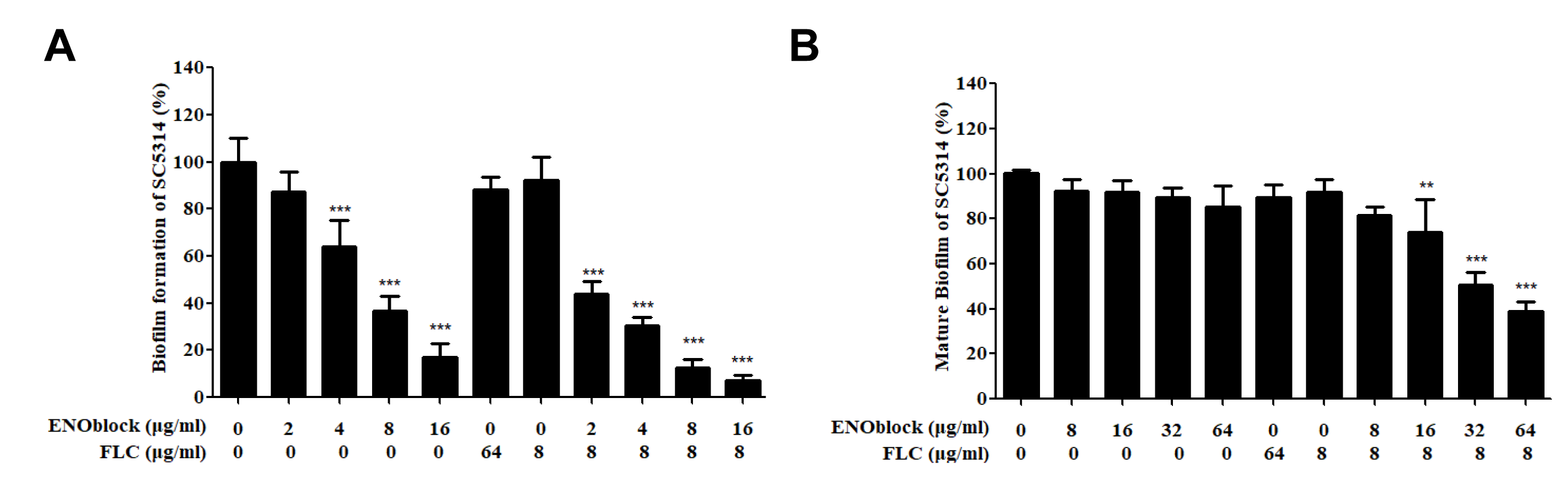

Supplement: FIGURE S3 — Effects of the combination of ENOblock and FLC on biofilm formation of C. albicans SC5314 in vitro. (A) Effects of different concentrations of ENOblock alone and in combination with 8 μg/ml FLC on C. albicans biofilm formation. (B) Effects of different concentrations of ENOblock alone and in combination with 8 μg/ml FLC on the maintenance of mature biofilms. Biofilm formation was evaluated by the XTT reduction assay by calculating the percentage of viable C. albicans cells relative to the control cells without drug treatment. Data are shown as the means ± standard deviations for three independent experiments. ∗∗P < 0.01; ∗∗∗P < 0.001 compared with the value of the control biofilms. [file Image_3.TIF]

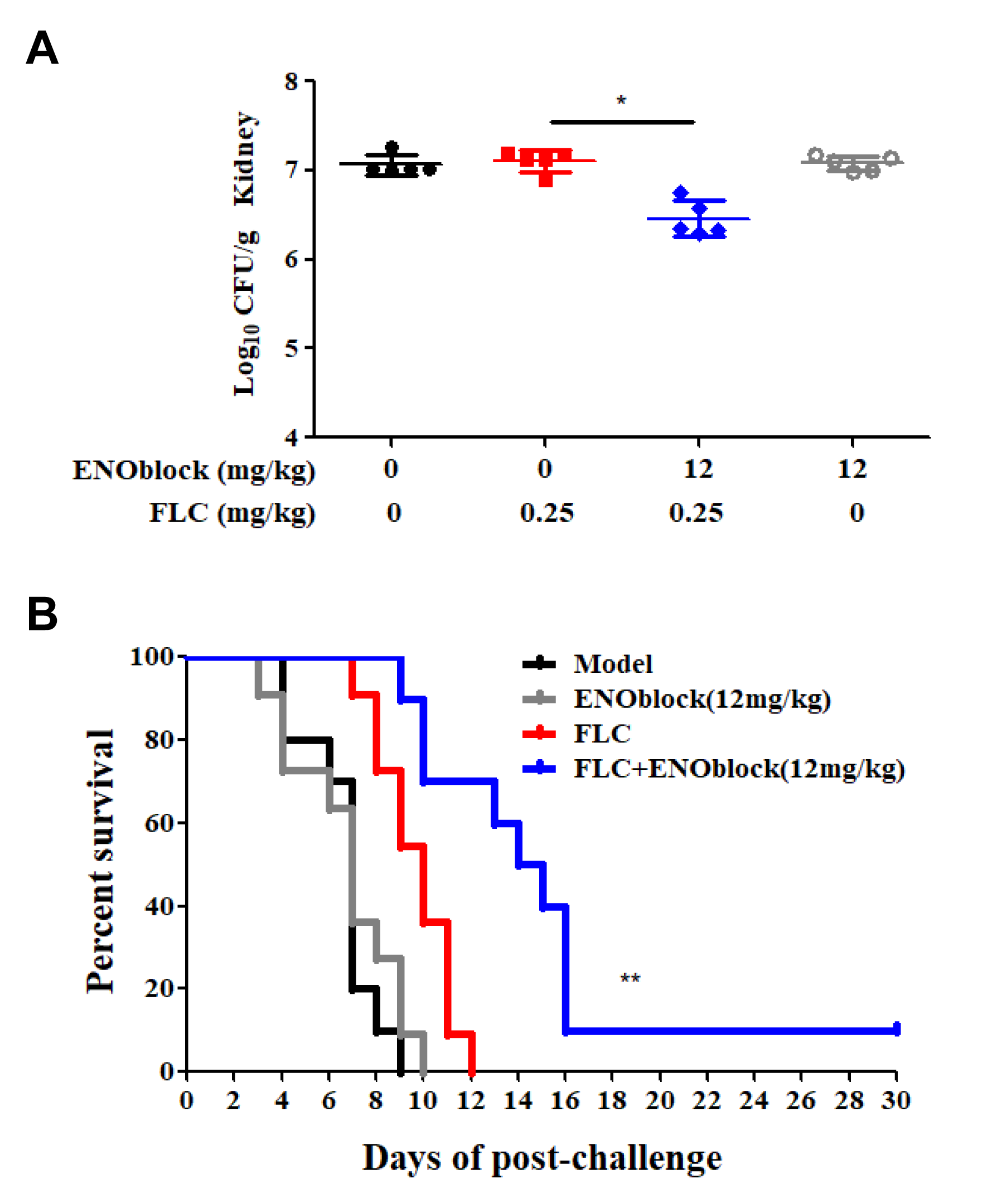

Supplement: FIGURE S4 — ENOblock enhances the activity of FLC in a murine model of systemic candidiasis. (A) Kidney CFU assay in mice with systemic candidiasis after 2 days. Female C57BL/6 mice were infected with 5 × 10^5^ CFU of C. albicans SC5314. ENOblock and FLC were administered 2 h post infection. ∗P < 0.05 (P-values are from ANOVA). (B) Survival curves of C57BL/6 mice infected with 5 × 10^5^ CFU of C. albicans SC5314. ENOblock and FLC were administered at 2, 24, and 48 h post infection. The log-rank test was used for statistical analysis. [file Image_4.TIF]

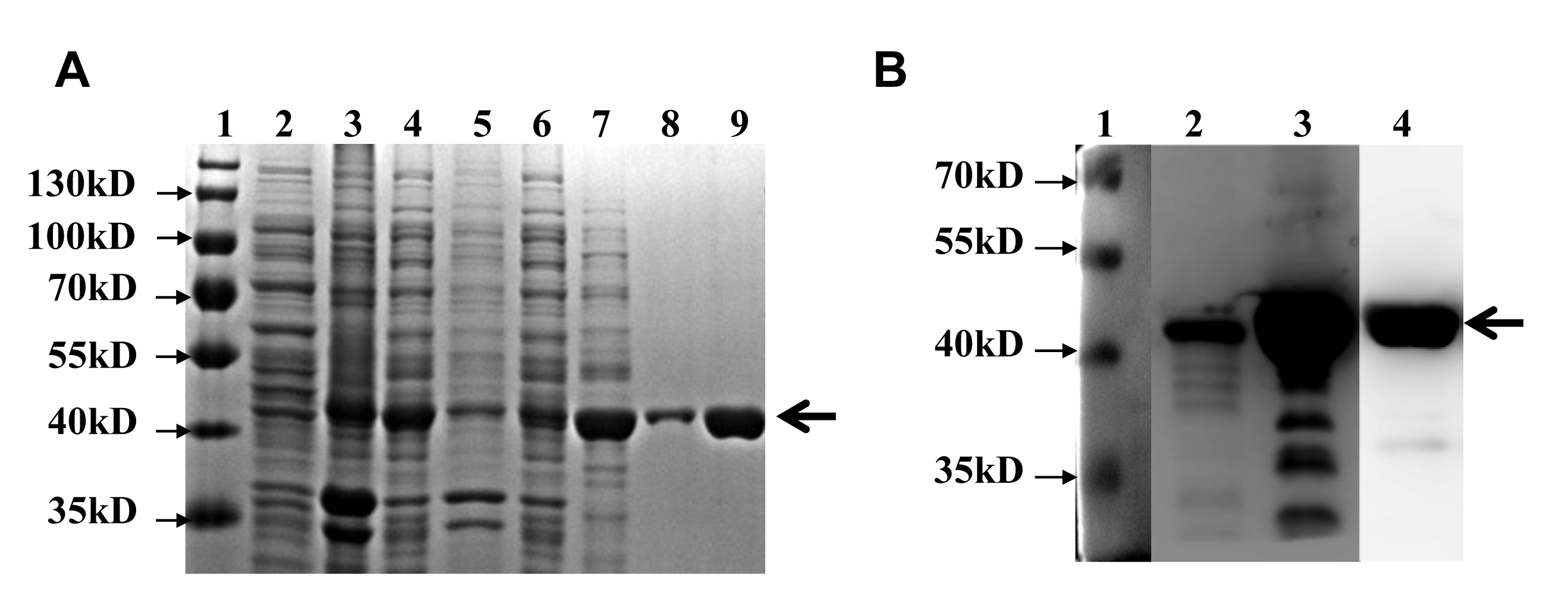

Supplement: FIGURE S5 — Expression and purification of recombinant C. albicans enolase 1 (rCaEno1). (A) The rCaEno1 protein was purified by Ni2+-NTA-agarose column chromatography as described, and elution fractions were evaluated by 10% SDS-PAGE. Lane 1, protein marker; lane 2, negative control (empty vector); lane 3, whole-cell protein; lane 4, supernatant fractions; lane 5, precipitation fractions; lanes 6–8, washed fractions; lane 9, purified rCaEno1 fusion protein. (B) Western blot of total proteins from C. albicans SC5314 (lane 2) and purified rCaEno1 protein (lane 3) using mouse anti-rCaEno1 protein antibodies and anti-His-tag polyclonal antibodies (lane 4). [file Image_5.TIF]

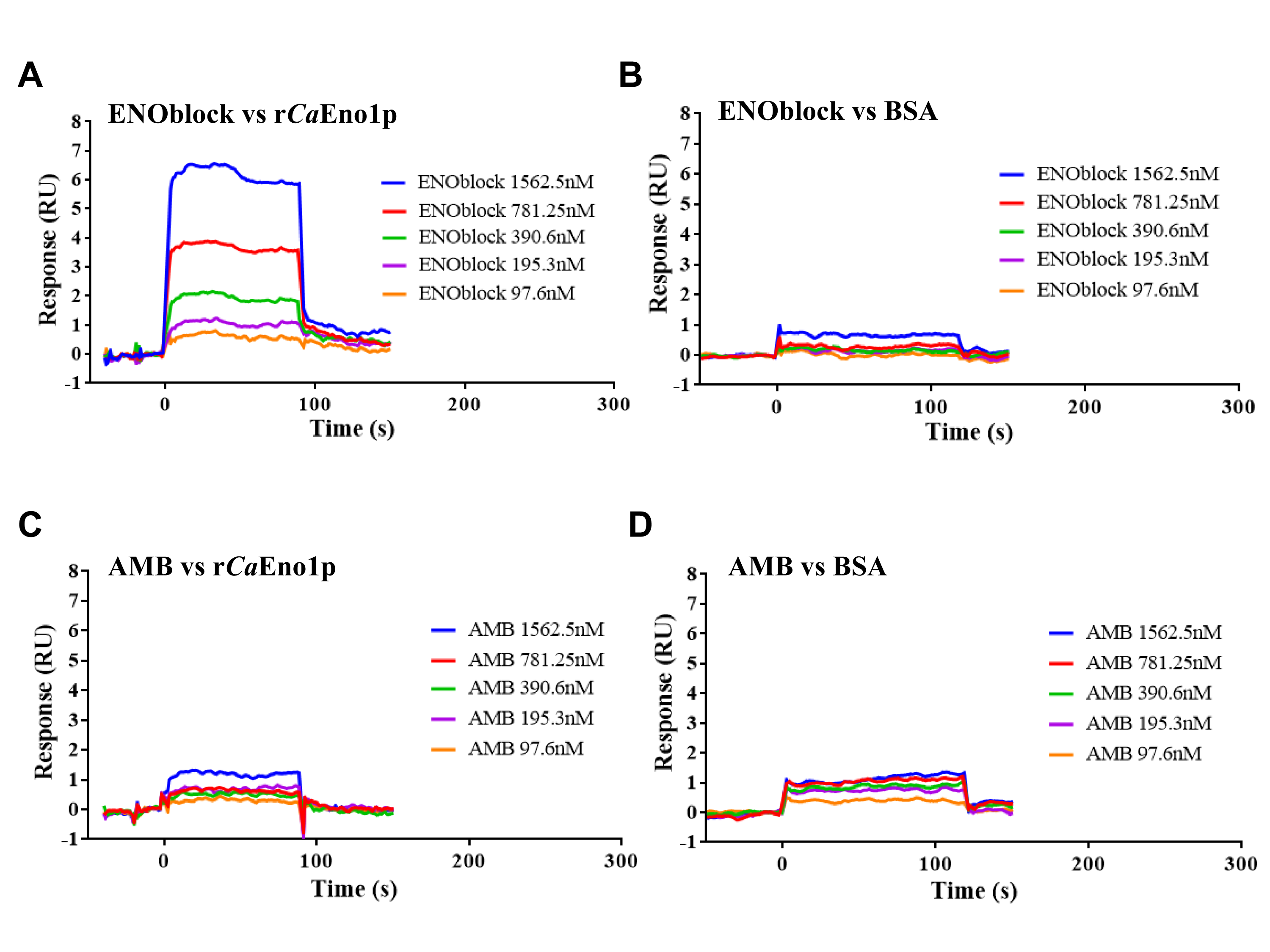

Supplement: FIGURE S6 — The interactions of rCaEno1 with ENOblock and amphotericin B measured by surface plasmon resonance. The rCaEno1 protein (BSA as the negative control) was coated on the CM5 sensor chip and serial dilutions (1562.5, 781.25, 390.6, 195.3, and 97.6 nM) of ENOblock (AMB as the control) were used as analytes. Changes in plasmon resonance are shown as response units. The panels (A,B) show binding curves (colored lines) obtained by passing different concentrations of ENOblock (1562.5, 781.25, 390.6, 195.3, and 97.6 nM) over rCaEno1 or BSA immobilized on a biosensor surface. The panels (C,D) show binding curves (colored lines) obtained by passing different concentrations of AMB (1562.5, 781.25, 390.6, 195.3, and 97.6 nM) over rCaEno1 or BSA immobilized on a biosensor surface. The data are representative of two independent experiments. AMB, amphotericin B. [file Image_6.TIF]
