# Supplementary material for: The Synergism of the Small Molecule ENOblock and Fluconazole Against Fluconazole-Resistant Candida albicans
Source: Front Microbiol. 2019 Sep 6;10:2071. doi: 10.3389/fmicb.2019.02071 (PMC6742966; doi:10.3389/fmicb.2019.02071)
Supplement: TABLE S1 — Interaction of fluconazole (FLC) and ENOblock against 30 FLC-resistant C. albicans. [file Table_1.DOCX]

| **Table S1 \|** Interaction of fluconazole (FLC) and ENOblock against 30 FLC-Resistant *C.albicans* | | | | | | |
| --- | --- | --- | --- | --- | --- | --- |
| **Clinical Isolates** | **MICs (µg/ml) alone** | |  | **MICs (µg/ml) in combination** | | **FICI** |
|  | **FLC** | **ENOblock** |  | **FLC** | **ENOblock** |  |
| *C. albicans* 0304103 | >64 | 32 |  | 2 | 8 | 0.266 |
| 385 | >64 | 32 |  | 1 | 8 | 0.258 |
| 395 | >64 | 32 |  | 2 | 4 | 0.141 |
| 538 | >64 | 32 |  | 8 | 8 | 0.313 |
| 540 | >64 | 32 |  | 1 | 8 | 0.258 |
| 12 | >64 | 32 |  | 1 | 8 | 0.258 |
| 14 | >64 | 32 |  | 2 | 4 | 0.141 |
| 100 | >64 | 32 |  | 1 | 8 | 0.258 |
| 379 | >64 | 32 |  | 0.5 | 8 | 0.254 |
| 953 | >64 | 32 |  | 1 | 8 | 0.258 |
| 1221 | >64 | 32 |  | 2 | 4 | 0.141 |
| 01010 | >64 | 32 |  | 1 | 8 | 0.258 |
| 0512681 | >64 | 16 |  | 16 | 4 | 0.375 |
| 305 | >64 | 32 |  | 8 | 4 | 0.188 |
| 17 | >64 | 32 |  | 0.5 | 8 | 0.254 |
| 646 | >64 | 32 |  | 1 | 4 | 0.133 |
| da2196 | >64 | 32 |  | 2 | 8 | 0.266 |
| 819 | >64 | 32 |  | 4 | 4 | 0.156 |
| 0710513 | >64 | 32 |  | 1 | 4 | 0.133 |
| 241 | >64 | 32 |  | 2 | 8 | 0.266 |
| 249 | >64 | 32 |  | 2 | 8 | 0.266 |
| 10231 | >64 | 32 |  | 0.5 | 8 | 0.254 |
| 5740 | >64 | 32 |  | 0.5 | 8 | 0.254 |
| 0605141 | >64 | 32 |  | 8 | 4 | 0.188 |
| 0710419 | >64 | 32 |  | 1 | 8 | 0.258 |
| 0512644 | >64 | 32 |  | 4 | 4 | 0.156 |
| 0710452 | >64 | 16 |  | 32 | 2 | 0.375 |
| 0710492 | >64 | 32 |  | 1 | 8 | 0.258 |
| 64548 | >64 | 32 |  | 1 | 8 | 0.258 |
| CAF2-1 | >64 | 16 |  | 4 | 4 | 0.281 |
|  |  |  |  |  |  |  |
