# Supplementary material for: The Synergism of the Small Molecule ENOblock and Fluconazole Against Fluconazole-Resistant Candida albicans
Source: Front Microbiol. 2019 Sep 6;10:2071. doi: 10.3389/fmicb.2019.02071 (PMC6742966; doi:10.3389/fmicb.2019.02071)
Supplement: TABLE S2 — Interaction of fluconazole (FLC) and ENOblock against 10 FLC-sensitive C. albicans. [file Table_2.DOCX]

| **Table S2 \|** Interaction of fluconazole (FLC) and ENOblock against 10 FLC-sensitive *C.albicans* | | | | | | |
| --- | --- | --- | --- | --- | --- | --- |
| **Clinical Isolates** | **MICs (µg/ml) alone** | |  | **MICs (µg/ml) in combination** | | **FICI** |
|  | **FLC** | **ENOblock** |  | **FLC** | **ENOblock** |  |
| *C. albicans* SC5314 | 1 | 32 |  | 1 | 1 | 1.03 |
| Y0109 | 0.5 | 16 |  | 0.25 | 1 | 0.56 |
| 9 | 1 | 16 |  | 1 | 1 | 1.06 |
| 10 | 0.5 | 16 |  | 0.5 | 1 | 1.06 |
| 11 | 0.5 | 32 |  | 0.5 | 1 | 1.03 |
| 28 | 0.5 | 32 |  | 0.25 | 1 | 0.75 |
| 465 | 0.5 | 32 |  | 0.5 | 1 | 1.03 |
| 805 | 1 | 32 |  | 1 | 1 | 1.03 |
| 0710253 | 0.5 | 32 |  | 0.5 | 1 | 1.03 |
| 0710502 | 0.5 | 32 |  | 0.5 | 1 | 1.03 |
|  |  |  |  |  |  |  |
